# Polymer/oxide bilayer dielectric for hysteresis-minimized 1 V operating 2D TMD transistors†

**DOI:** 10.1039/c7ra12641g

**Published:** 2018-01-12

**Authors:** Minho Yoon, Kyeong Rok Ko, Sung-Wook Min, Seongil Im

**Affiliations:** Institute of Physics and Applied Physics, Yonsei University 50 Yonsei-ro, Seodaemun-gu Seoul 120-749 Korea semicon@yonsei.ac.kr +82-2-392-1592 +82-2-2123-2842

## Abstract

Despite their huge impact on future electronics, two-dimensional (2D) dichalcogenide semiconductor (TMD) based transistors suffer from the hysteretic characteristics induced by the defect traps located at the dielectric/TMD channel interface. Here, we introduce a hydroxyl-group free organic dielectric divinyl-tetramethyldisiloxane-bis (benzocyclobutene) (BCB) between the channel and conventional SiO_2_ dielectric, to practically resolve such issues. Our results demonstrate that the electrical hysteresis in the n-channel MoS_2_ and p-channel MoTe_2_ transistors were significantly reduced to less than ∼20% of initial value after being treated with hydrophobic BCB dielectric while their mobilities increased by factor of two. Such improvements are certainly attributed to the use of the hydroxyl-group free organic dielectric, since high density interface traps are related to hydroxyl-groups located on hydrophilic SiO_2_. This concept of interface trap reduction is extended to stable low voltage operation in 2D MoTe_2_ FET with 30 nm BCB/10 nm Al_2_O_3_ bilayer dielectric, which operates well at 1 V. We conclude that the interface engineering employing the BCB dielectric offers practical benefits for the high performance and stable operation of TMD-based transistors brightening the future of 2D TMD electronics.

## Abstract 2

By inserting hydroxyl-group free organic dielectric between hydrophilic oxide dielectric and 2D TMD channel, highly stable 2D FETs are achieved. This concept was successfully extended to a practical device application such as stable 1 V operation of 2D MoTe_2_ FET.

## Graphical abstract

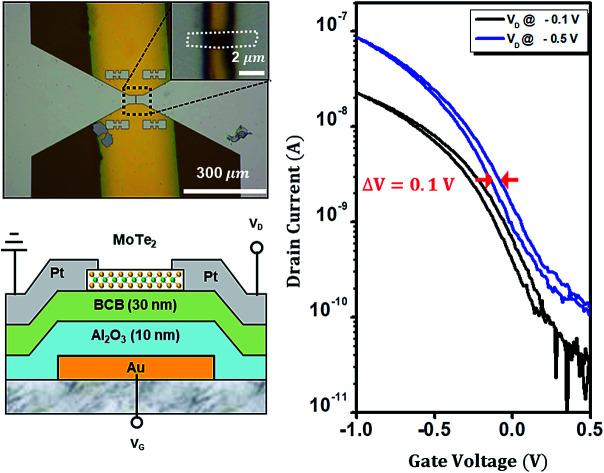

## Introduction

Two-dimensional (2D) dichalcogenide semiconductors (TMD) have been extensively studied during recent years due to their massive potential for next-generation electronics.^1,2^ Compared to the gapless semiconductor graphene,^3^ TMD such as molybdenum disulfide (MoS_2_),^4^ molybdenum ditelluride (MoTe_2_),^5^ and tungsten diselenide (WSe_2_)^6^ provide the tunable electronic bandgap which is dependent on the thickness of the layer itself. Due to their discrete bandgap, the TMD-based field effect transistors (FETs) often exhibit clear switching operation with its relatively high values of mobility and ON/OFF current ratio.^7–10^ However, several reports reveal that the electrical performances of the devices are significantly degraded by the interface and surface states of the devices.^11,12^ For a good candidate to minimize the interfacial defect states in FET, the hydrophobic hexagonal boron nitride (h-BN) as dielectric and passivation layer has been proposed.^13,14^ Although this approach has been encouraging, h-BN is basically expensive due to its special growth processes for good crystalline quality control,^15,16^ and furthermore, additional elaborate processes are still requested for h-BN flake to be incorporated in device fabrications. Hence, alternatives to replace h-BN have been suggested: self-assembled monolayers,^17^ organic insulating materials,^18^ high-k oxide dielectrics,^19^ and *etc.* Among these candidates, an organic insulating polymer, divinyl-tetramethyldisiloxane-bis(benzocyclobutene) (BCB) could be an appropriate option for TMD-channel FETs. BCB is a well-known non-polar organic polymer with hydroxyl-group free chemical structure and it has been widely used as a dielectric for stable high-performance organic transistors.^20–22^ To the best of our limited knowledge, BCB has never been attempted for the TMD-based field effect transistors while it is easily formed by spin-casting.

In the present study, we report n-channel MoS_2_ and p-channel MoTe_2_ transistors with the BCB gate dielectric on oxide gate dielectric. According to our results, initial hysteresis in the MoS_2_ and MoTe_2_ transistors on 285 nm-thick SiO_2_/p^+^-Si back gate without BCB were ∼15 V in their transfer curve characteristics, but it reduced to ∼4 V in the devices with BCB. Based on these hysteresis minimization effects by BCB, we successfully extended our results to a more practical device application, 1 V operation of MoTe_2_ FET with 30 nm-thin BCB on 10 nm-thin atomic layer deposited (ALD) Al_2_O_3_. It is thus regarded that BCB dielectric layer offers benefits for the high performance and stable operation of TMD-based transistors. The main advantage of the BCB would be its non-polar hydrophobicity and process conveniences.

## Experimental detail

For basic investigations on 2D TMD device stability, 300 nm-thick organic insulating material, divinyl-tetramethyldisiloxane-bis (benzocyclobutene) (BCB, CYCLOTENE, Dow Chemical) was formed on the 285 nm-thick SiO_2_/p^+^-Si substrate by spin-casting and subsequent thermal annealing at 300 °C for 10 minutes in nitrogen ambient. (But for extended practical device fabrication, 30 nm-thin BCB on 10 nm-thin atomic layer deposited Al_2_O_3_ was formed on patterned Au gate). Then, MoS_2_ and MoTe_2_ flakes for n and p-type transistors were micromechanically exfoliated with polydimethylsiloxane (PDMS) stamps, respectively. Those TMD flakes are subsequently dry-transferred onto the two types of dielectric substrates: BCB/SiO_2_ and SiO_2_. Then, 50 nm-thick Au for MoS_2_ and 100 nm-thick Pt for MoTe_2_ were sputter-deposited on the substrates for source/drain electrodes and patterned by conventional lift-off processes. The thickness of the flakes were confirmed with the atomic force microscopy (AFM) and also identified with Raman spectroscopy.

The current–voltage (*I*–*V*) measurements of the transistors were performed with a semiconductor parameter analyser (Model HP4155C, Agilent Technologies) and the capacitance–voltage (*C*–*V*) measurements of the devices were conducted with a LCR meter (Model HP4284A, Agilent Technologies). Electrical characterisations of the devices were carried out entirely in air ambient (relative humidity RH ∼ 45%) at room temperature, but for one MoTe_2_ device sample was also measured in vacuum (less than 1 Torr) at 300 K, to investigate the hysteresis-induction effects of air molecules adsorbed on TMD channel surface.

## Results and discussion

**Fig. 1:**
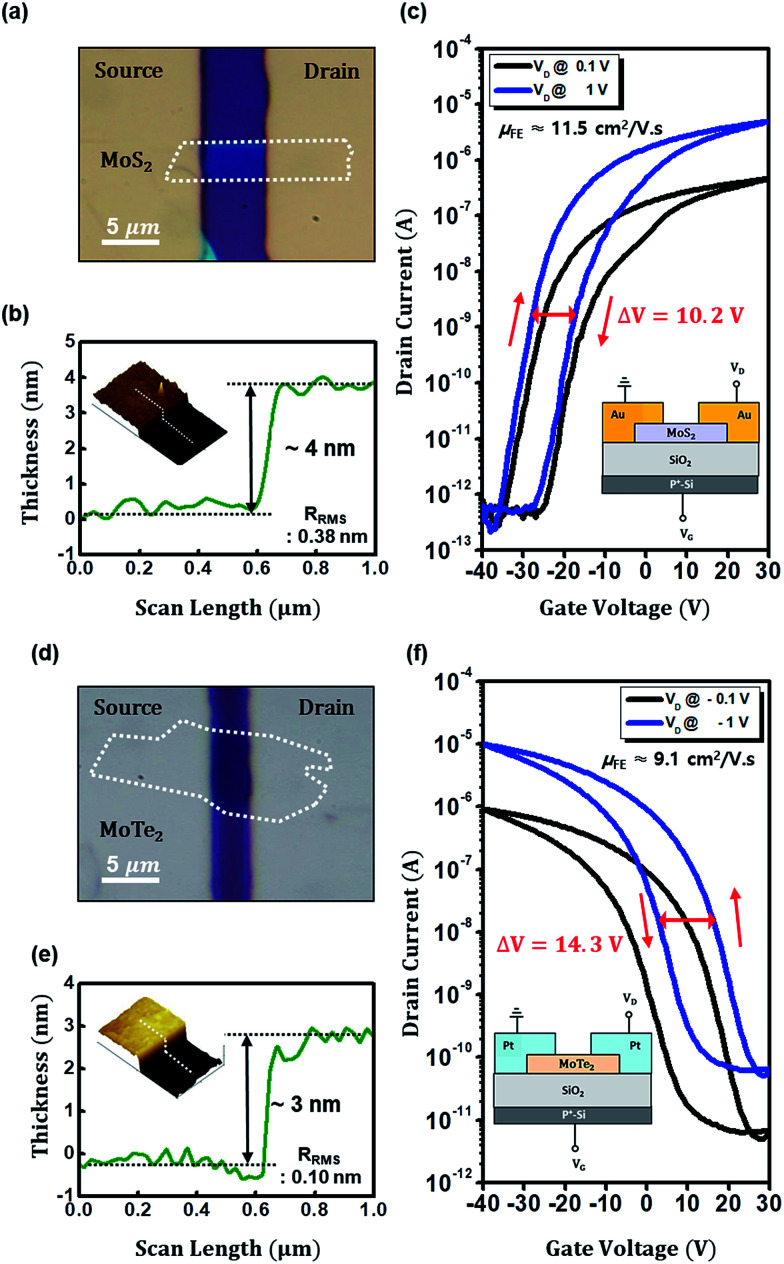
(a–c) Optical microscopy (OM) image, AFM image along with channel thickness profiles (∼4 nm), and transfer characteristics (*I*_DS_*vs. V*_GS_) of n-type MoS_2_ FET on 285 nm-thick SiO_2_ dielectric with cross section scheme (inset). The channel width and length of the MoS_2_ FET are 4.1 and 5.4 μm, respectively, and the device shows the electron mobility of 11.5 cm^2^ V^−1^ s^−1^. The value of the clockwise hysteresis is around 10.2 V. (d–f) OM image, AFM image along with thickness profiles (∼3 nm), and the transfer characteristics of p-type MoTe_2_ FET on 285 nm-thick SiO_2_ dielectric with cross section scheme (inset). The channel width and length of the MoTe_2_ FET are 8.1 and 3.4 μm, respectively, and the device shows the hole mobility of cm^2^ V^−1^ s^−1^. The value of the anticlockwise hysteresis is around 14.3 V.

Fig. 1a and d show bottom-gate top-contact MoS_2_ and MoTe_2_ transistors on 285 nm thick SiO_2_ dielectric, respectively. Their thickness appears to be 4 nm for MoS_2_ and 3 nm for MoTe_2_ as measured by atomic force microscopy (AFM) scan in Fig. 1b and e. Identity of the two flakes was again confirmed by Raman spectroscopy whose results are seen in ESI section (Fig. S1†). Fig. 1c and f show the transfer characteristics (*I*_DS_*vs. V*_GS_) of our bottom-gate top-contact MoS_2_ and MoTe_2_ transistors, respectively. Their insets are schematic cross sections of those devices. The MoS_2_ transistor with Au source/drain electrodes displays n-type conduction behaviour with the electron mobility of 11.5 cm^2^ V^−1^ s^−1^ and high on/off current ratio (>10^5^). The MoTe_2_ transistor with Pt source/drain electrodes presents p-type conduction with the hole mobility of 9.1 cm^2^ V^−1^ s^−1^ and high on/off current ratio (>10^5^) as well. Although the electrical performances of the devices are promising as reported previously by others,^23,24^ it is very apparent that the devices much suffer from the gate bias-induced hysteresis (Δ*V* = ∼10.2 V for n-MoS_2_ and ∼14.3 V for p-MoTe_2_ FET) in the present transfer characteristics, which should be minimized for practical device applications in electronics.

Numerous reasons count for the hysteresis in TMD-based 2D transistors as discussed enormously in the organic and inorganic transistors for decades,^25–27^ and the reasons were classified into four main categories: the adsorption of water and oxygen molecules on semiconductor channel surface, defects in the semiconductor material, mobile trap charges in the dielectric, and the interface trap charges between the semiconductor and the contacting dielectric. Here, we mainly focused on the interface effects because the single-crystalline-like 2D semiconducting channel would meet hydrophilic oxide dielectric in general and their interface becomes to contain high density charge traps which originate from the surface hydroxyl-group of the oxides dielectric.^12,28^

To this end, a hydrophobic organic insulator, BCB was conceived as an excellent option for the dielectric for the transistors because the BCB film has a hydroxyl-group free chemical structure and can be deposited by simple spin-casting.

**Fig. 2:**
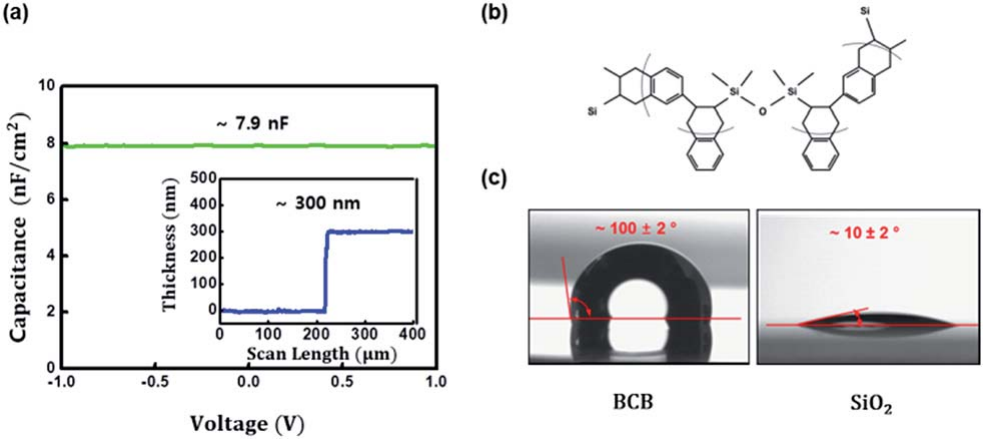
(a) The geometrical capacitance of the BCB dielectric in Au/BCB/Au structure with the thickness of BCB dielectric (inset). (b) Chemical structure of the BCB. (c) The contact angle of D.I. water on the BCB and SiO_2_, respectively. The contact angle of D.I. water on the hydrophobic BCB dielectric was ∼100° while that on a cleaned SiO_2_ hydrophilic substrate was ∼10°.

Prior to exploiting the BCB as a dielectric layer for MoS_2_ and MoTe_2_ transistors, the dielectric properties and hydrophobicity of the BCB layer were initially investigated. The capacitance of our BCB dielectric was measured to be ∼7.9 nF cm^−2^ at 1 kHz with top and bottom Au electrodes (Au/BCB/Au) in the Fig. 2a, where the thickness of the spin-casted BCB layer was ∼300 nm (inset of the Fig. 2a) as scanned with a surface profiler. Deduced dielectric constant of the BCB dielectric at the thickness was ∼2.67, which is in a good agreement with the previously reported value.^29^ Furthermore, as shown in the Fig. 2c, the contact angle of D.I. water on the BCB dielectric was ∼100° while that on a cleaned SiO_2_ substrate was as low as ∼10°; such contact angle measurements clearly indicate the hydrophobicity of the BCB layer surface due to its chemical structure (Fig. 2b).^30^

**Fig. 3:**
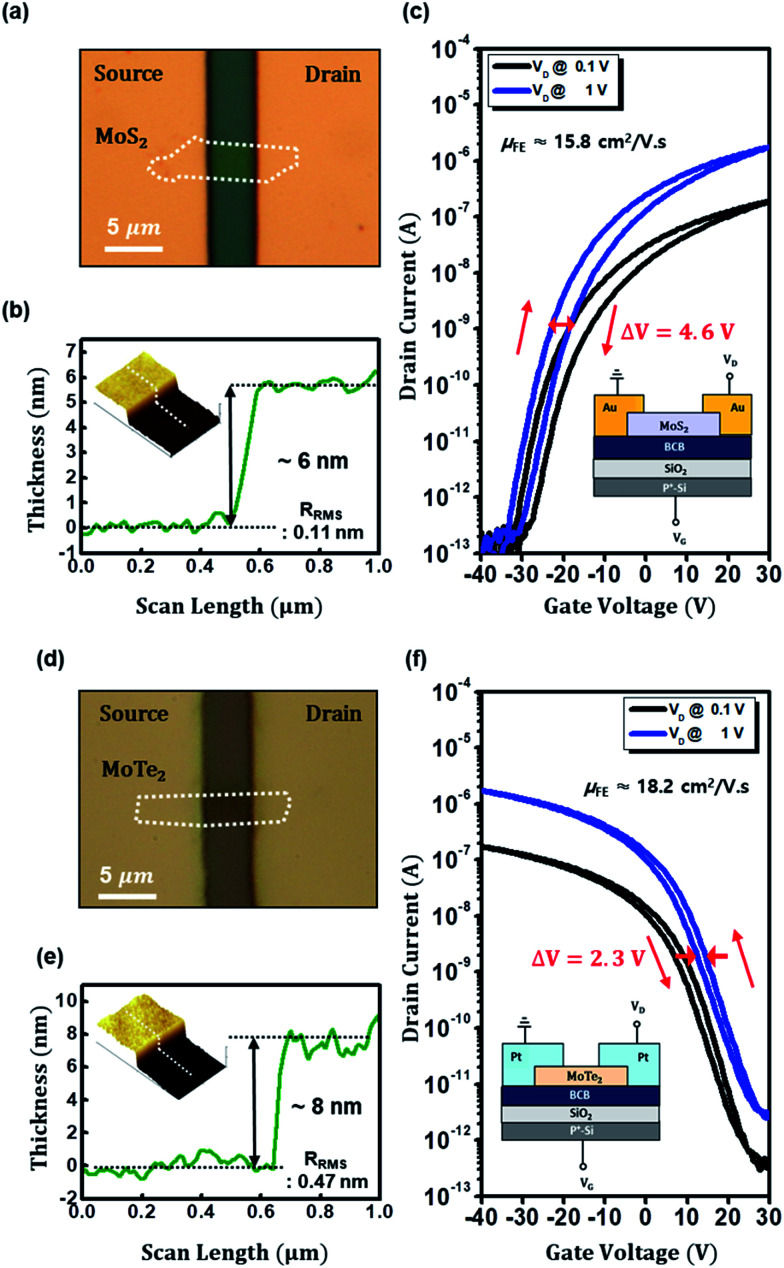
(a–c) Optical microscopy image, AFM image along with channel thickness profile (∼6 nm), and the transfer characteristics (*I*_DS_*vs. V*_GS_) of n-MoS_2_ FET with 300 nm-thick BCB/SiO_2_ dielectric with cross section scheme (inset). The channel width and length of the MoS_2_ FET are 2.5 and 2.9 μm, respectively, and the device shows the electron mobility of 15.8 cm^2^ V^−1^ s^−1^. The value of the clockwise hysteresis is around 4.6 V. (d–f) OM image, AFM image along with channel thickness profile (∼8 nm), and the transfer characteristics of p-MoTe_2_ FET with BCB/SiO_2_ dielectric with cross section scheme (inset). The channel width and length of the MoTe_2_ FET are 2.8 and 3.9 μm, respectively, and the device shows the hole mobility of cm^2^ V^−1^ s^−1^. The value of the anticlockwise hysteresis is around 2.3 V.

Fig. 3a and d show bottom-gate top contact MoS_2_ and MoTe_2_ transistors with BCB (300 nm)/SiO_2_ dielectric, respectively. Their thickness appears to be 6 nm for MoS_2_ and 8 nm for MoTe_2_ as measured by atomic force microscopy (AFM) scan in Fig. 3b and e. Fig. 3c and f show the transfer characteristics (*I*_DS_–*V*_GS_) of the bottom-gate top contact MoS_2_ and MoTe_2_ transistors on the BCB/SiO_2_ dielectric, respectively. Their insets are schematic cross sections of those devices. With BCB dielectric, the values of the hysteresis in the MoS_2_ and MoTe_2_ transistors were dramatically reduced to ∼4.5 V and ∼2.3 V. (Similar hysteresis reduction is also shown in the output characteristics of Fig. S2†). Furthermore, the field-effect mobility of the devices was significantly increased by the factor of two in both cases (from 11.5 to 15.8 cm^2^ V^−1^ s^−1^ in MoS_2_ transistors and from 9.1 to 18.2 cm^2^ V^−1^ s^−1^ in MoTe_2_ transistors). We thus regard that the BCB dielectric effectively minimizes the trap sites or passivate the electro-active hydroxyl groups at the dielectric/TMD channel interface improving the electrical performance and the stability of the devices. On the one hand, the linear mobility of our FETs was estimated with the following equation.^31^1
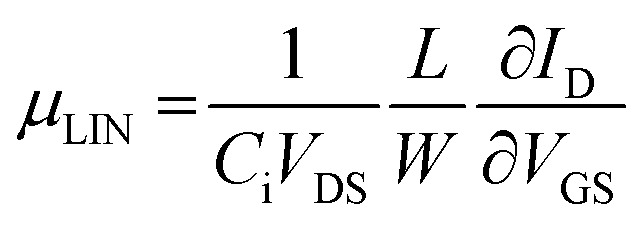
where *C*_i_ is the geometric dielectric capacitance, *L* is channel length and *W* is its width.

Based on aforementioned transfer characteristics from TMD-devices with and without BCB dielectric, we considered quantifying the effective interface trap density, *D*_it_ by deducing the values from the subthreshold swing (SS) of the transistors, which is expressed in the following equation.^31^2
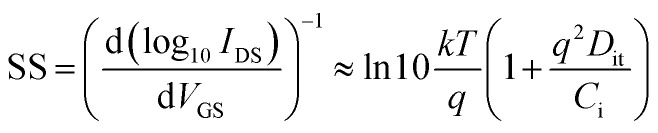
where *k* is the Boltzmann constant, *T* is the temperature in Kelvin, *q* is the electronic charge, and *C*_i_ is the geometric capacitance of the dielectric.

We thought this type of approach is at least worthy even though this SS equation ignores any effects caused by contact resistance which is sometimes not ignorable at all.^32^ The subthreshold swing (SS) values of the MoS_2_ transistors with BCB/SiO_2_ and SiO_2_ dielectrics seem similar each other as about 2.2 V dec^−1^ and 2.0 V dec^−1^, respectively, but their geometric capacitances are quite different, to be 4.8 nF cm^−2^ and 12.1 nF cm^−2^. Thus, the estimated values of *D*_it_ become 1.1 × 10^12^ cm^−2^ eV^−1^ and 2.4 × 10^12^ cm^−2^ eV^−1^ for the n-MoS_2_ transistors with and without BCB. Similarly, the SS values of p-MoTe_2_ transistors with and without BCB dielectric were 3.6 V dec^−1^ and 3.4 V dec^−1^, resulting in *D*_it_ values of 1.9 × 10^12^ cm^−2^ eV^−1^ and 4.4 × 10^12^ cm^−2^ eV^−1^, respectively. Likewise, devices with BCB dielectric appear to contain 2–3 times lower number density of traps at the interface in SS-based estimation. However, such SS-based approach and *D*_it_ results must be still unclear because SS behaviour cannot ignore contact resistance effects from TMD/source-drain electrode contact.^33^ In addition, since the gate hysteresis of *I*_DS_–*V*_GS_ transfer characteristics (*I*–*V* measurement system) unavoidably contains source/drain contact resistance effects in general.^34^ Hence, it might be necessary to find other measurement scheme which is immune from contact effects.

**Fig. 4:**
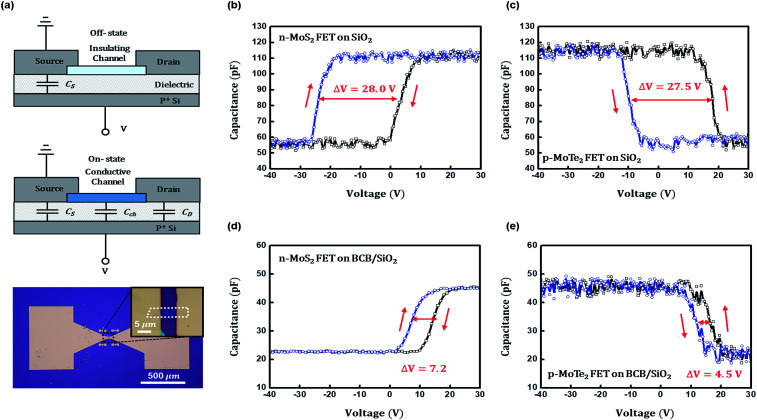
(a) Schematic circuit and illustration of the modified *C*–*V* measurements on our TMD FETs. In channel depletion, a capacitance (*C*_S_) is measured from the dielectric area under only one electrode (source), however such capacitance is doubled-up by channel accumulation (note the plots in (b–e)), which would connect the source and drain [the other electrode for *C*_D_ (=*C*_S_)]. The channel area (∼less than 25 μm^2^ as seen in the inset OM) for *C*_ch_ is incomparably smaller than that of one electrode which is around 4.55 × 10^5^ μm^2^. According to the *C*–*V* characteristics obtained from (b) MoS_2_ on SiO_2_, (c) MoTe_2_ on SiO_2_, (d) MoS_2_ on BCB/SiO_2_, and (e) MoTe_2_ on BCB/SiO_2_, the initial voltage hysteresis is dramatically reduced from ∼28 V to 4.5–7.2 V due to the BCB effects.

We thus conceived to perform capacitance–voltage (*C*–*V*) measurements for evaluating the trapped interface charges, because *C*–*V* measurements are oriented to focus on the interface by gate bias without any interference from the contact resistance effects.^35^ For the *C*–*V* measurements on FET structure at high and low frequencies, DC and small signal AC voltages are applied to the gate electrode while source/drain (S/D) electrodes are grounded in general.^36^ However, general *C*–*V* method on FET structure would not be effective if the channel area is too small compared to the S/D-to-gate overlap area; real capacitance signals from channel should be overridden by parasitic capacitance. Our device with small 2D channel would definitely meet such parasitic capacitance issue, so we modified the general *C*–*V* measurements by grounding the source electrode only, considering that DC voltage sweep would eventually induce the capacitance of channel and drain electrode area by channel accumulation or channel conducting.^15^ Since the trapped charges cannot respond at high frequency, the interface trap density can be extracted at a low frequency of 100 Hz, using the sample transistor architecture as shown in Fig. 4a. According to the schematic circuits of Fig. 4a, we meet with two cases during DC sweep: channel depletion and accumulation which are dependent on the DC bias. In the case of channel depletion, a capacitance (*C*_S_) is measured from the dielectric area under only one electrode (source), however such capacitance should be doubled-up by channel accumulation, which would connect the source and drain (the other electrode; now total capacitance becomes *C*_S_ + *C*_D_ = 2*C*_S_). According to the *C*–*V* plots of Fig. 4b–e, the capacitance value is indeed doubled-up from 55 to 112 pF (for FETs without BCB but SiO_2_) or from 22 to 45 pF (for FETs with BCB). In our device (photo images of Fig. 1a and 4a), the channel area for channel capacitance (*C*_ch_) is too small (incomparable to electrode area) to be visibly counted for total capacitance. In spite of that, the TMD channels successfully performed as connecting/disconnecting (on/off) switches.

Noticeable in Fig. 4b and c is that those TMD channels clearly show hysteresis from forward-to-backward sweep. Because trapping and de-trapping of charges at the interface are certainly related to the forward and backward sweep, respectively, it is anticipated that the interfacial trap density would be quite precisely estimated using the hysteresis voltage. For both n-MoS_2_ and p-MoTe_2_ FETs with only SiO_2_ dielectrics, almost the same amount of large voltage hysteresis was obtained to be 27.5–28 V in Fig. 4b and c, respectively. The hysteresis was significantly reduced to be 7.2 and 4.5 V of for n- and p-FETs as seen in Fig. 4d and e. This result is readily expected from the related transfer curves in Fig. 3c and f. Small amount of hysteresis still remains as 4.5–7.2 V due to the small trap density at the BCB dielectric/TMD channel interface. But such number is only about 15–25% of previous trap density on SiO_2_. Based on our *C*–*V* results, it is very likely that the hysteresis observed from *I*_DS_–*V*_GS_ transfer characteristics is mainly from gate dielectric/channel interface, not from the TMD surface effects involved with air molecules. For further confirmation on the dielectric/channel interface-induced hysteresis, we subsidiary performed *I*_DS_–*V*_GS_ transfer characteristics of another MoTe_2_ device with BCB dielectric in vacuum ambient at 300 K. As shown in Fig. S3 of ESI,† already-reduced hysteresis by BCB application seems not decreased any further even in vacuum, which supports our assumption that such hysteresis is mainly related to the amount of interfacial traps.

With the results from *C*–*V* characteristics in Fig. 4a–e, we could easily estimate the interfacial trap densities of MoS_2_ and MoTe_2_-based FETs with and without BCB, using the following simple equation, *Q*_it_ = *C*_i_Δ*V*/*q*, where Δ*V* is the voltage hysteresis and *C*_i_ is the geometric capacitance (Farad cm^−2^) of the dielectric. *C*_i_ value can be obtained from the *C*–*V* curves in Fig. 4b and d because we already know the electrode area. From the equation, the estimated values of the interface trap charge density were 2.08 × 10^12^ cm^−2^ eV^−1^ and 2.11 × 10^12^ cm^−2^ eV^−1^ in the MoS_2_ and MoTe_2_ transistors on SiO_2_ dielectric. But those values were an order of magnitude reduced to the values of 2.2 × 10^11^ cm^−2^ eV^−1^ and 1.4 × 10^11^ cm^−2^ eV^−1^ when BCB was inserted as a dielectric layer. Table 1 summarizes all the values on interface trap densities and device performances. Here, we assumed that the trap charges only stem from the dielectric/channel interface, and the trap density values by SS and *D*_it_ measurement were worked out as overall average trap density (*D*_it_ × energy gap).^24^ According to Table 1, SS-driven method always results in higher values of trap density than the values by *C*–*V* method whether the device has BCB dielectric or not, because any *I*–*V* method implicitly reflects the contact resistance effects.^34^ The difference between SS-driven and *C*–*V* methods is very clear in the FETs with BCB dielectric, but such difference becomes relatively quite small in the other devices with high density traps (with only SiO_2_ dielectric).

**Table 1:** Summary of the mobility, subthreshold swing, hysteresis, and estimated trap densities of the devices

Semicon.	Dielec.	Capacit. (nF cm^−2^)	Mobility (cm^2^ V^−1^ s^−1^)	S.S (V dec^−1^)	Hysteresis (Δ*V*) from		Trap density (×10^12^ cm^−2^) from	
					Transfer curves	Modified *C*–*V*	S.S	Modified *C*–*V*
MoS_2_	SiO_2_	12.1	11.5	2.0 ± 0.1	10.2 ± 0.1	28.0 ± 0.1	2.9 ± 0.1	2.12 ± 0.01
MoS_2_	BCB/SiO_2_	4.8	15.8	2.2 ± 0.1	4.6 ± 0.1	7.2 ± 0.1	1.30 ± 0.03	0.22
MoTe_2_	SiO_2_	12.1	9.1	3.4 ± 0.1	14.3 ± 0.1	27.5 ± 0.1	4.4 ± 0.1	2.08 ± 0.01
MoTe_2_	BCB/SiO_2_	4.8	18.2	3.6 ± 0.1	2.3 ± 0.1	4.5 ± 0.1	1.86 ± 0.03	0.14

**Fig. 5:**
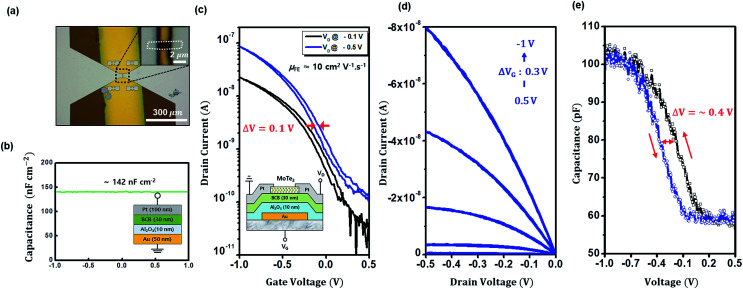
(a) Optical image of our MoTe_2_ FET with BCB/Al_2_O_3_ dielectric and Au bottom gate, along with inset magnified image of 5 nm-thin MoTe_2_ channel (*W*/*L* ratio ∼ 1 μm/2 μm = 0.5). (b) The geometrical capacitance of the thin BCB/Al_2_O_3_ dielectric. (Inset: schematic illustration of device cross section) (c) transfer characteristics of MoTe_2_ FET. ON/OFF *I*_D_ current ratio appears to be ∼1000. (Inset: schematic illustration of device cross section. Au gate was patterned on thick SiO_2_/p^+^-Si wafer substrate). (d) Output characteristics show a very low operational voltage of 0.5–1 V. (f) *C*–*V* curve hysteresis voltage was measured, to be ∼0.4 V which leads to an estimated interface trap. density of ∼3.5 × 10^11^ cm^−2^.

Although we have mainly focused on minimizing the interfacial trap density in the present study, we also extended our results to a more practical device application as our final effort: low voltage operational MoTe_2_ FET with 30 nm-thin BCB on 10 nm-thin atomic layer deposited (ALD) Al_2_O_3_. Fig. 5a and its inset show optical images of our MoTe_2_ FET with BCB/Al_2_O_3_ dielectric and Au bottom gate, where width-to-length ratio of the device was 1 μm/2 μm. Capacitance of BCB/Al_2_O_3_ was ∼142 nF cm^−2^ as obtained from metal–insulator–metal (MIM) *C*–*V* measurement (Fig. 5b). We also confirmed the BCB thickness of 30 nm with a mechanical profiler in Fig. S4a† and the MoTe_2_ thickness of 5 nm by AFM scan (Fig. S4b†). From the transfer characteristics, device mobility (linear regime) turned out to be ∼10 cm^2^ V^−1^ s^−1^, and ON/OFF *I*_D_ current ratio appears to be ∼1000 (Fig. 5c). Transfer and output characteristics (Fig. 5c and d) show a very low operational voltage of 0.5–1 V. The *C*–*V* curve hysteresis was measured in Fig. 5e, to be ∼0.4 V which leads to an estimated interface trap density of ∼3.5 × 10^11^ cm^−2^. Since the trap density is comparable to those from thick BCB/SiO_2_ in Table 1, it is well regarded that 30 nm-thin BCB polymer on 10 nm Al_2_O_3_ keeps the function of trap minimization ensuring the practical low voltage operation as well. Such hysteresis-reduced low voltage operation in 2D TMD FET has rarely been demonstrated.^17,37^

## Conclusions

We have fabricated a few layer n-MoS_2_ and p-MoTe_2_ channel FETs with and without BCB dielectric on SiO_2_/p^+^-Si. Our results from *C*–*V* measurements and *I*–*V* transfer characteristics display that the hysteresis in the MoS_2_ and MoTe_2_ transistors were significantly reduced to less than ∼20% of initial value after treated with hydrophobic BCB dielectric, while the linear mobilities of both p- and n-FETs increased by factor of two. Such improvements are certainly attributed to the hydroxyl-group free organic dielectric, BCB on SiO_2_, since high density interface traps are related to hydroxyl-groups located on SiO_2_. In particular, our modified *C*–*V* measurements turned out to be a more useful tool than *I*–*V* characteristics for the quantification of interface trap density. Our concept of interface trap reduction was successfully applied to stable low voltage operation in 2D MoTe_2_ FET with 30 nm BCB/10 nm Al_2_O_3_ bilayer dielectric. We thus conclude that the interface engineering employing the BCB dielectric offers practical benefits for the high performance and stable operation of TMD-based transistors and brightens the future of 2D TMD electronics.

## Conflicts of interest

There are no conflicts to declare.

## Supplementary Material

RA-008-C7RA12641G-s001

## References

[cit1] Jariwala D., Sangwan V. K., Lauhon L. J., Marks T. J., Hersam M. C. (2014). ACS Nano.

[cit2] Wang Q. H., Kalantar-zadeh K., Kis A., Coleman J. N., Strano M. S. (2012). Nat. Nanotechnol..

[cit3] Novoselov K. S., Geim A. K., Morozov S. V., Jiang D., Katsnelson M. I., Grigorieva I. V., Dubonos S. V., Firsov A. A. (2005). Nature.

[cit4] Mak K. F., Lee C., Hone J., Shan J., Heinz T. F. (2010). Phys. Rev. Lett..

[cit5] Lezama I. G., Arora A., Ubaldini A., Barreteau C., Giannini E., Potemski M., Morpurgo A. F. (2015). Nano Lett..

[cit6] Desai S. B., Seol G., Kang J. S., Fang H., Battaglia C., Kapadia R., Ager J. W., Guo J., Javey A. (2014). Nano Lett..

[cit7] Chuang H.-J., Tan X., Ghimire N. J., Perera M. M., Chamlagain B., Cheng M. M.-C., Yan J., Mandrus D., Tománek D., Zhou Z. (2014). Nano Lett..

[cit8] Kang K., Xie S., Huang L., Han Y., Huang P. Y., Mak K. F., Kim C.-J., Muller D., Park J. (2015). Nature.

[cit9] Shokouh S. H. H., Jeon P. J., Pezeshki A., Choi K., Lee H. S., Kim J. S., Park E. Y., Im S. (2015). Adv. Funct. Mater..

[cit10] Kim S., Konar A., Hwang W.-S., Lee J. H., Lee J., Yang J., Jung C., Kim H., Yoo J.-B., Choi J.-Y., Jin Y. W., Lee S. Y., Jena D., Choi W., Kim K. (2012). Nat. Commun..

[cit11] Shu J. P., Wu G. T., Guo Y., Liu B., Wei X. L., Chen Q. (2016). Nanoscale.

[cit12] Chan M. Y., Komatsu K., Li S.-L., Xu Y., Darmawan P., Kuramochi H., Nakaharai S., Aparecido-Ferreira A., Watanabe K., Taniguchi T., Tsukagoshi K. (2013). Nanoscale.

[cit13] Lee G.-H., Cui X., Kim Y. D., Arefe G., Zhang X., Lee C., Ye F., Watanabe K., Taniguchi T., Kim P., Hone J. (2015). ACS Nano.

[cit14] Cui X., Lee G.-H., Kim Y. D., Arefe G., Huang P. Y., Lee C.-H., Chenet D. A., Zhang X., Wang L., Ye F., Pizzocchero F., Jessen B. S., Watanabe K., Taniguchi T., Muller D. A., Low T., Kim P., Hone J. (2015). Nat. Nanotechnol..

[cit15] Kim S. M., Hsu A., Park M. H., Chae S. H., Yun S. J., Lee J. S., Cho D.-H., Fang W., Lee C., Palacios T., Dresselhaus M., Kim K. K., Lee Y. H., Kong J. (2015). Nat. Commun..

[cit16] Lee K. H., Shin H., Lee J., Lee I., Kim G., Choi J., Kim S. (2012). Nano Lett..

[cit17] Kawanago T., Oda S. (2016). Appl. Phys. Lett..

[cit18] Bao W., Cai X., Kim D., Sridhara K., Fuhrer M. S. (2013). Appl. Phys. Lett..

[cit19] Chang H. Y., Yang S., Lee J., Tao L., Hwang W. S., Jena D., Lu N., Akinwande D. (2013). ACS Nano.

[cit20] Chua L., Zaumseil J., Chang J., Ou E. C.-W., Ho P. K.-H., Sirringhaus H., Friend R. H. (2005). Nature.

[cit21] Anthopoulos T. D., Singh B., Marjanovic N., Sariciftci N. S., Montaigne Ramil A., Sitter H., Cölle M., De Leeuw D. M. (2006). Appl. Phys. Lett..

[cit22] Saudari S. R., Frail P. R., Kagan C. R. (2009). Appl. Phys. Lett..

[cit23] Lee H. S., Min S.-W., Chang Y.-G., Park M. K., Nam T., Kim H., Kim J. H., Ryu S., Im S. (2012). Nano Lett..

[cit24] Choi K., Lee Y. T., Kim J. S., Min S. W., Cho Y., Pezeshki A., Hwang D. K., Im S. (2016). Adv. Funct. Mater..

[cit25] Veres J., Ogier S., Lloyd G. (2004). Chem. Mater..

[cit26] Sirringhaus H. (2009). Adv. Mater..

[cit27] Jeong J. K. (2011). Semicond. Sci. Technol..

[cit28] Guo Y., Wei X., Shu J., Liu B., Yin J., Guan C., Han Y., Gao S., Chen Q. (2015). Appl. Phys. Lett..

[cit29] Chua L. L., Ho P. K. H., Sirringhaus H., Friend R. H. (2004). Appl. Phys. Lett..

[cit30] Kong L., Cheng Y., Jin Y., Ren Z., Li Y., Xiao F. (2015). J. Mater. Chem. C.

[cit31] Klauk H. (2010). Chem. Soc. Rev..

[cit32] Lee J. S., Chang S., Bouzid H., Koo S. M., Lee S. Y. (2010). Phys. Status Solidi A.

[cit33] Choi K., Raza S. R. A., Lee H. S., Jeon P. J., Pezeshki A., Min S., Kim J. S., Yoon W., Ju S., Lee K., Im S. (2015). Nanoscale.

[cit34] Bittle E. G., Basham J. I., Jackson T. N., Jurchescu O. D., Gundlach D. J. (2016). Nat. Commun..

[cit35] Kimura M. (2011). Solid-State Electron..

[cit36] Cohen N. L., Paulsen R. E., White M. H. (1995). IEEE Trans. Electron Devices.

[cit37] Lee H. S., Baik S. S., Lee K., Min S., Jeon P. J., Kim J. S., Choi K., Choi H. J., Kim J. H., Im S. (2015). ACS Nano.

