# Supplementary material for: Polymer/oxide bilayer dielectric for hysteresis-minimized 1 V operating 2D TMD transistors
Source: RSC Adv. 2018 Jan 12;8(6):2837–43. doi: 10.1039/c7ra12641g (PMC9077681; doi:10.1039/c7ra12641g)
Supplement: RA-008-C7RA12641G-s001 [file RA-008-C7RA12641G-s001.pdf]

Electronic Supplementary Material (ESI) for RSC Advances.
This journal is © The Royal Society of Chemistry 2018

Supporting Information

Polymer/Oxide Bilayer Dielectric for Hysteresis-Minimized 1 V Operating 2D TMD Transistors

*Minho Yoon, Kyeong Rok Ko, Sung-Wook Min and Seongil Im**

Department of Physics, Yonsei University, Seoul 120-749, Korea

Corresponding Author

*E-mail: semicon@yonsei.ac.kr

S1. Identification of MoS_2 and $MoTe_2$ flakes

As shown in Figure S1a, The MoS_2 flakes are identified with the Raman spectroscopy analysis, where the vibrational peaks at 382.3 cm^{-1} and 382.6 cm^{-1} correspond to the in-plane (E^1_{2g}) modes while those at 406.6 cm^{-1} and 404.9 cm^{-1} are attributed to the out of plane (A_{1g}).[1] Analogously, the $MoTe_2$ flakes are also confirmed with the same Raman analysis (Figure S1b), where the vibrational peaks at 174.0 cm^{-1} and 176.3 cm^{-1} are related to the out of plane modes of A_{1g} and the peaks of 240.4 cm^{-1} and 240.4 cm^{-1} are to B^1_{2g} while those at 240.4 cm^{-1} and 240.4 cm^{-1} are for the in-plane (E^1_{2g}) modes, respectively.[2]

S2. The output characteristics of our bottom-gate top-contact MoS_2 and $MoTe_2$ FETs with and without BCB dielectric on SiO_2 /p^+-Si.

Figure S2 (a-d) display the 2 the output characteristics of our bottom-gate top-contact MoS_2 and $MoTe_2$ FETs with and without BCB dielectric on SiO_2 /p^+-Si. All the devices clearly operate in the linear regime without any contact issues.

S3. Study the surface effect on the gate-induced hysteresis on the TMD based FETs

In an effort to study the surface effect on the gate-induced hysteresis on the TMD based FETs, transfer characteristics (I_D vs.V_{GS}) of the bottom-gate top contact $MoTe_2$ transistors on the BCB/SiO_2 dielectric are investigated in air ambient and under vacuum. As shown in the Figure S3 (b), the values of the voltage hysteresis in both condition appear to be almost same values of ~ 5 V, although the turn-on voltage of the device are shifted. Therefore, we consider the interface trap density is the dominant reason for the voltage hysteresis in the transfer curves.

S4. Ultra-thin BCB layer for low-voltage operation and identification of the $MoTe_2$ flake

For a more practical device application, the ultra-thin BCB layer from the diluted solution (4 wt%, *CYCLOTENE* in Mesitylene) was formed on the substrate and the thickness was identified as 30 nm with a surface profiler as shown in Figure S4 (a). Following, we fabricated the low voltage operational $MoTe_2$ FET with 30 nm-thin BCB on 10 nm-thin atomic layer deposited (ALD) Al_2O_3. The thickness of the $MoTe_2$ Flake appears to be 5 nm by atomic force microscopy (AFM) scan in Figure S4 (b).

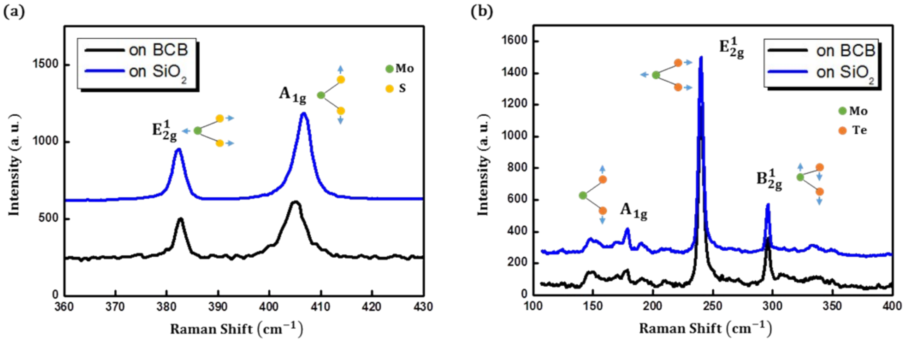

Figure S1. Raman shift of (a) MoS_2 on SiO_2 and BCB dielectrics and the spectra of (b) $MoTe_2$ on SiO_2 and BCB dielectrics. No difference has been found whether TMDs are on BCB or on SiO_2.

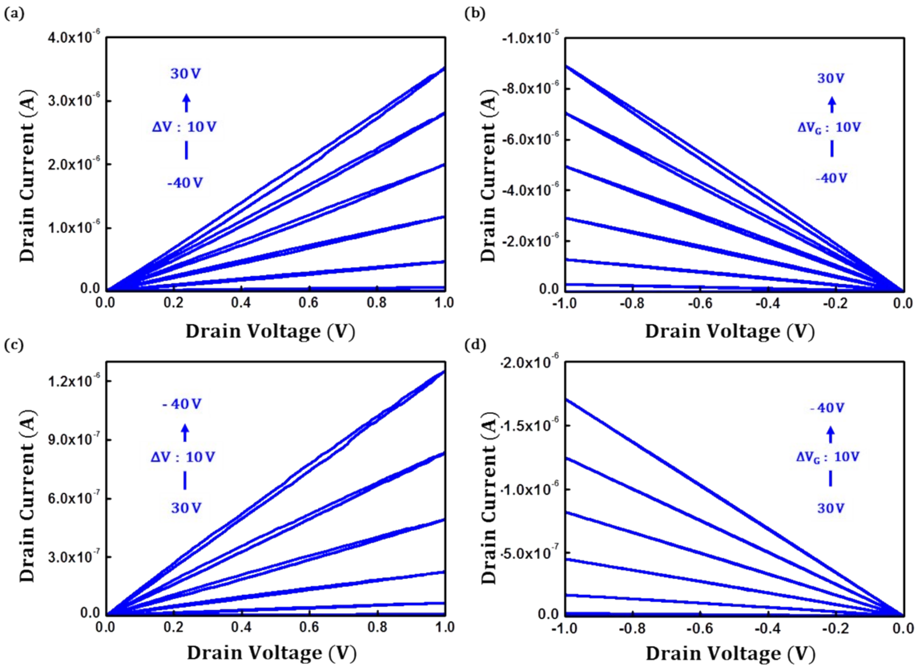

Figure S2. Output characteristics of the (a) MoS_2 FET on SiO_2, (b) $MoTe_2$ FET on SiO_2, (c) MoS_2 FET on BCB/SiO_2, and (d) $MoTe_2$ FET on BCB/SiO_2, respectively. All the devices clearly operate in the linear regime.

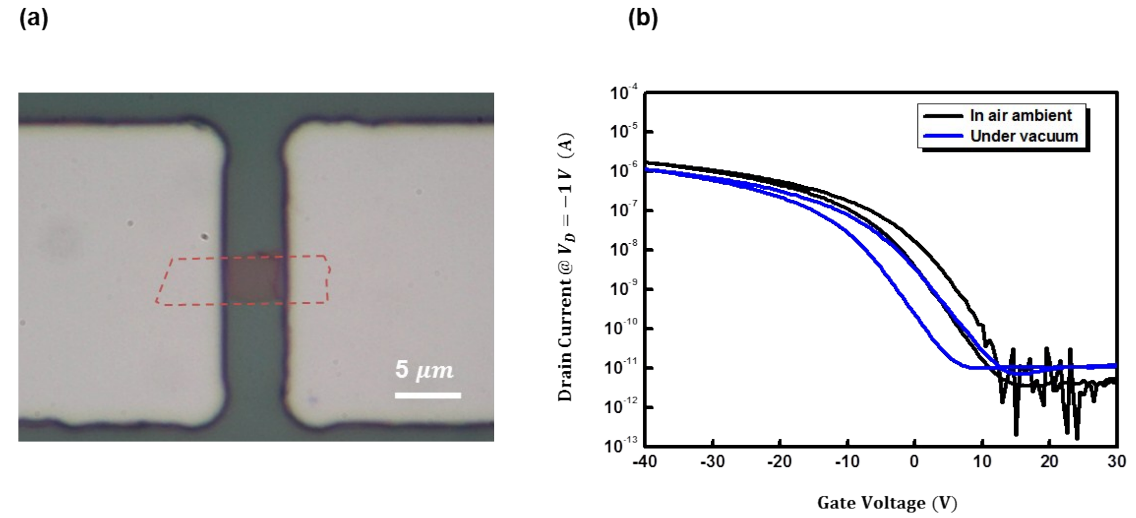

Figure S3. (a) Optical microscopy (OM) images of the bottom-gate top-contact $MoTe_2$ FET on BCB/SiO_2 dielectric, where the channel width and length are 3.3 and 4.9 μm, respectively. (b) Transfer characteristics (I_D Vs. V_{GS}) of the device in air ambient and under vacuum at 300 K.

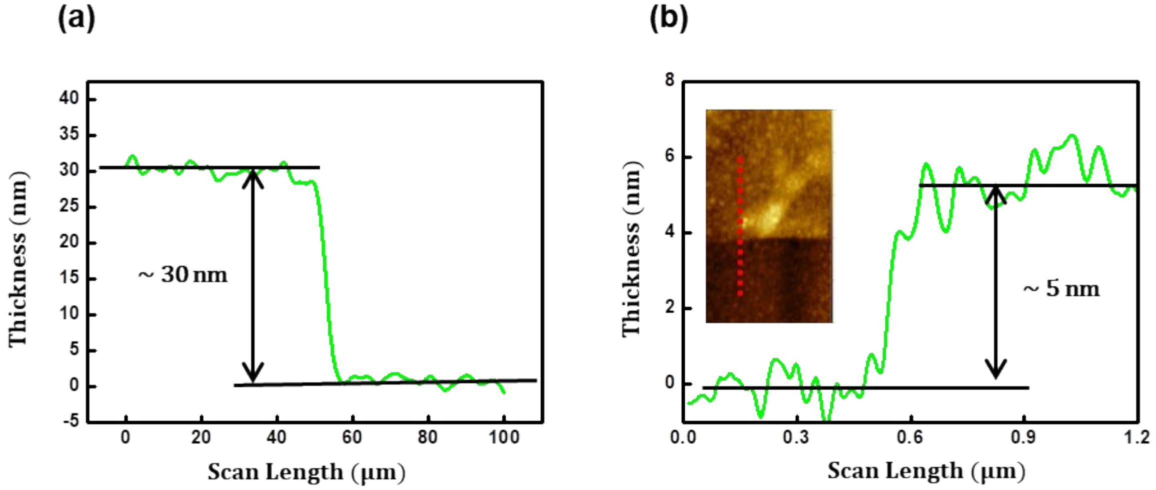

Figure S4. (a) The BCB thickness of 30 nm was confirmed with a mechanical profiler. (b) The AFM image along with channel thickness profile (~ 5 nm) of our $MoTe_2$ FET with BCB/Al_2O_3 dielectric.

References

1 H. Li, Q. Zhang, C. C. R. Yap, B. K. Tay, T. H. T. Edwin, A. Olivier and D. Baillargeat, *Adv. Funct. Mater.*, 2012, **22**, 1385–1390.

2 M. Yamamoto, S. T. Wang, M. Ni, Y. F. Lin, S. L. Li, S. Aikawa, W. Bin Jian, K. Ueno, K. Wakabayashi and K. Tsukagoshi, *ACS Nano*, 2014, **8**, 3895–3903.
